# Retrospective analysis of longitudinal melanonychia: A Chinese experience

**DOI:** 10.3389/fped.2022.1065758

**Published:** 2023-01-16

**Authors:** Anqi Lyu, Yinglong Hou, Qiying Wang

**Affiliations:** ^1^ Department of Dermatology, Xiang'an Hospital of Xiamen University, School of Medicine, Xiamen University, Xiamen, China; ^2^ Department of Plastic Surgery, The First Affiliated Hospital of Zhengzhou University, Zhengzhou, China

**Keywords:** Hutchinson's sign, longitudinal melanonychia, nail matrix nevus, subungual melanoma, retrospective study

## Abstract

**Objective:**

We aimed to analyze the clinical and histopathologic characteristics of longitudinal melanonychia (LM), explore the differences between adults and children, and propose some recommendations.

**Methods:**

Data on pigmentation, lentigo, subungual melanoma (SUM), and nail matrix nevus (NMN) were acquired for comparison.

**Results:**

Lesions on thumbs in the children’s group were significantly fewer (*p* = 0.006) than in adults. Lesions on little fingers in children were more than in adults; the difference was statistically significant (*p *= 0.025). The widths of bands in adults were wider than in children (*p* < 0.001), and the duration and width were positively correlated (*r* = 0.474). There was more pigmentation in adults than in children; the difference was statistically significant (*p* = 0.005). NMN was reported in 56.1% children and 34.3% adults; the difference was statistically significant (*p* = 0.005). Adults had six SUM cases, whereas none in children; the difference was statistically significant (*p* = 0.006). The recurrence rate in adults was significantly higher than in children (*p* = 0.019).

**Conclusion:**

The widths of bands increase with the course, indicating that LM may be progressive. The four pathological types have different distributions with age, and each type requires different treatment. The lower recurrence rate in children suggests that LM needs diagnosis and appropriate treatment as soon as possible.

## Introduction

Longitudinal melanonychia (LM) is longitudinal pigmentation on a fingernail or toenail. It can be gray, brown, or black. LM may appear on more than one finger. It can be caused by fungi, bacteria, nail matrix lentigo, nevi of the nail matrix, hyperplasia, benign activation of melanocytes (1), or traumatic nail disorders (2, 3). The width ranges from a thin band to covering the nail, and the clinical feature is variable. LM diagnosis is based on a thorough history, clinical features, and fungal and bacterial cultures (4). It remains a challenge for dermatologists, and histological examination remains the gold standard (3).

Melanocytic activation and hyperpigmentation of the nail matrix epithelium are common causes of LM in adults (5). Four histologic types explain LM entities: pigmentation, inflammation, nail matrix nevus (NMN), and subungual melanoma (SUM). All but SUM are benign lesions. Clinically, NMN produces regular longitudinal brown or black streaks without worrying features. In children, these features may appear in benign lesions (2).

NMN is the most common type in children with LM (6) and sometimes exhibits clinical features similar to SUM. Some features of NMN resemble acral nevi. In the early stage, freckled structures of melanocytes with irregular cell nests are often seen. Melanocytes may present above the basal layer. It is a worrying feature that melanocytes are found in the nail plate (7). The nail fold epithelium may also be involved.

SUM arises from the nail matrix, appears as a black line in the early stage, and gradually infects the entire nail (8). The general characteristics are Hutchinson's sign, nail plate deformity, and irregular pigmentation pattern (5, 9, 10). Hutchinson's sign is the spread of pigment seen on the adjacent skin and proximal/lateral/distal nail folds. It is considered a risk indicator, indicating the growth of nail melanoma. If present, excisional histopathology should be performed to exclude nail melanoma. SUM accounts for 6% of adult LMs (11) and 1%–3% of melanoma in the white race (12). Some studies report that 19%–40% of acral melanoma is SUM (13–16). Thumb, index finger, and hallux are the most affected (17, 18). SUM diagnosis remains a challenge even for specialists, especially in early lesions. The diagnostic criteria include poor circumscription, density of melanocytes, irregular distribution of melanocytes, cytologic atypia, and lymphocytic infiltrate (7, 19, 20). The differences between SUM and NMN lie in uneven color, blurred boundaries, and expanding trends.

In some cases, dermatoscopy may be a tool for the diagnosis of benign or malignant lesions (6). However, nail plate dermatoscopy fails to find the source of pigment in nail matrix epithelium (20). Melanocytes and melanin pigments are difficult to discern; thus, Fontana staining is required to identify melanin, and immunostaining is used to quantify melanocytes. Due to the poor prognosis of some LM types, LM distinction between children and adults remains unclear, and no consistent treatment principles have been developed. More precise delineation of their characteristics is required. In this retrospective study, we explored the differences between adult and pediatric LM and made some recommendations.

## Methods

This was a retrospective study on Chinese patients in a single center. A total of 174 LM nails were treated in our hospital from June 2018 to May 2021. Patients were excluded for having trauma, a history of drug use, fungal melanocychia, or endocrine system disease. Inclusion criteria included clinical presentation, surgery of LM, and available follow-up information. All histopathological diagnoses were made independently by two pathologists (Li DM and Wang GN). Any differences in the assessment were resolved by a consensus conference.

Medical records of age, sex, site involved, color, duration, width, Hutchinson's sign, pathology, and follow-up were reviewed. The follow-up ranged from 12 to 36 months. The cases were divided into two groups: children under 18 years and adults above 18 years.

### Statistical methods

All statistical analyses were conducted by SPSS 21.0 (IBM Corp, Armonk, NY). The tests were conducted by Pearson's *χ*^2^. The *p* values less than 0.05 were considered statistically significant.

### Ethical information

The study was approved by the Review Board of the First Affiliated Hospital of Zhengzhou University, Henan, China. The patients were informed fully, there was no interference in diagnosis and treatment, and medical records were collected. All patients signed the written informed consent stating that the information was used only for research (2018-LW-024).

## Results

### Clinical characteristics

**Figure 1:**
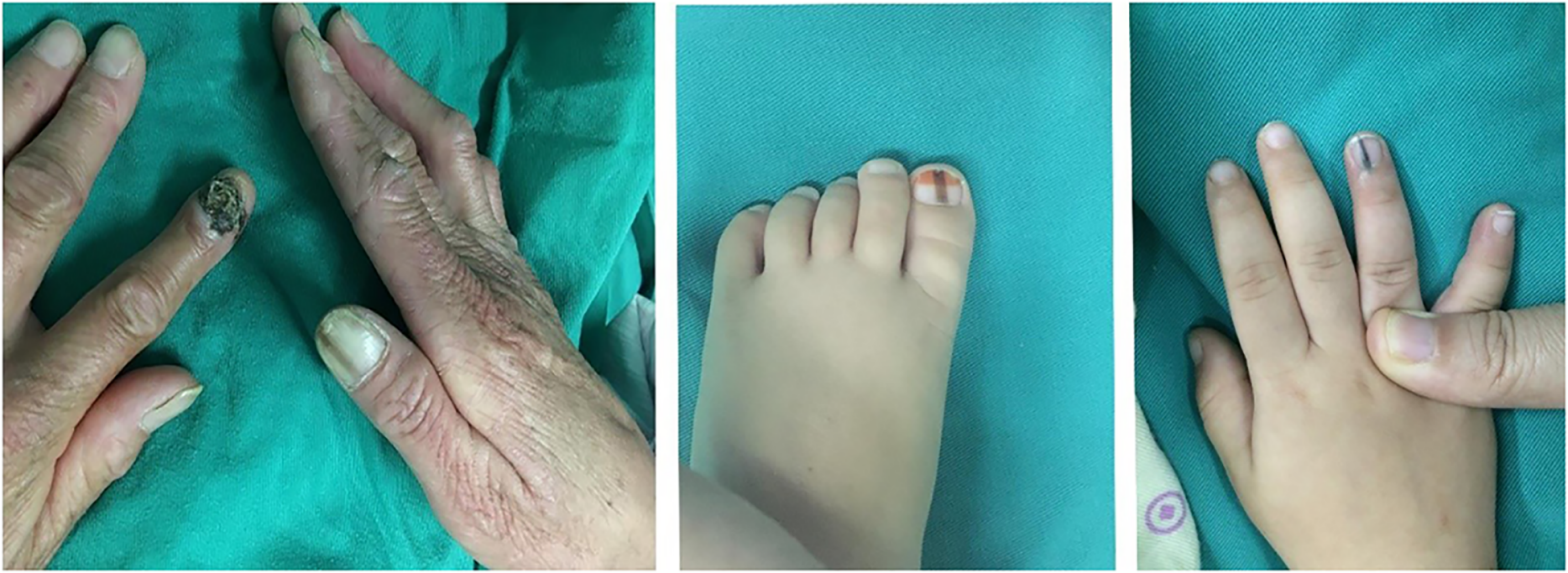
A woman and one boy had two lesions, respectively.

A total of 174 pigmented nails from 172 patients (87 men, 85 women; mean age 21.6 years) were assessed. Two cases had more than one lesion (Figure 1). One boy had one on the left hallux and another on the right ring finger. A woman had an SUM on the left index finger and an NMN on the right thumb. Table 1 lists the results. The course ranged from 1 month to 34 years, with an average of 43.66 months. The mean follow-up time was 11.76 months. The children’s group had 106 patients (56 boys, 50 girls; mean age 5.8 years), and the adults’ group had 66 patients (31 men, 35 women; mean age 37.6 years). The widths in adults were wider than in children (*p* < 0.001). The correlation coefficient between the width and course reached 0.474, and the significance level was 0.1. There was a significant positive correlation between them.

**Table 1:** Clinical data.

	Children (*n* = 106)	Adults (*n* = 66)	*p* Value
Age, years	5.8 ± 4.1	37.6 ± 14.6	
Course, months	27.9 ± 25.3	73.3 ± 92.8	
Follow-up, months	14.7 ± 3.1	13.9 ± 3.5	
Hutchinson's sign	18	11	
Sex			
Male	56	31	
Female	50	35	
Number of nails affected			
1	105	65	
2	1	1	
Width			<0.001
<3 mm	37	5	
≥3 mm	70	62	

The locations of all lesions are presented in Table 2. Thumbs were the most infected in both groups, but the proportions (29.9% vs. 50.7%) were different; it was considered statistically significant (*χ*^2^ = 7.600, *p *= 0.006). Little fingers in children were significantly more infected than in adults (13.1% > 3.0%, *χ*^2^ = 5.033, *p* = 0.025).

**Table 2:** Site data.

	Children (%)	Adults (%)	*p* Value
Thumb	32 (29.9)	34 (50.7)	0.006
Index finger	18 (16.8)	7 (10.4)	
Medius	8 (7.5)	6 (9.0)	
Ring finger	11 (10.3)	8 (12.0)	
Little finger	14 (13.1)	2 (3.0)	0.025
Hallux	15 (14.0)	7 (10.4)	
Other toes	9 (8.4)	3 (4.5)	

### Histopathologic analysis

LM was divided into four types according to histopathology: pigmentation, lentigo, NMN, and SUM. (1) Pigmentation means pigment in the cuticle or basal layer without nevus cells. (2) In lentigo, melanocytes proliferate but there is no cell nest. (3) In NMN, nevus cells are evident, forming cell nests. There are three types of nevi according to the location of the nevus cells: compound, junctional, and intradermal. (4) In SUM, malignant melanoma occurs under the nail.

Table 3 presents the numbers of respective types. The prevalence of pigmentation in the children’s group was significantly lower (38.3% < 53.7%) than that in the adults’ group (*χ*^2^ = 3.968, *p* = 0.046). The adults’ group had six SUM cases (9.0%), whereas none in children; the difference was statistically significant (*χ*^2^ = 7.417, *p =* 0.006). The percentage of NMN in children was higher (56.1% > 34.3%) than in adults; it was statistically significant (*χ*^2^ = 7.810, *p =* 0.005). In NMN cases, compound nevus accounted for 11.7% in children and 65.2% in adults; the difference was statistically significant (*χ*^2^ = 24.476, *p *< 0.001). The contribution of junctional nevus in children was significantly greater than in adults (68.3% > 8.7%, *χ*^2^* =* 23.685, *p *< 0.001). In the six SUM cases (Table 4), four cases had undergone a biopsy at their first visit without surgery. Later, they came to our hospital for resection after their conditions deteriorated. We suspect that biopsy stimulates the disease to become malignant.

**Table 3:** Pathological types.

	Children (%)	Adults (%)	*p* Value
Pigmentation	41 (38.3)	36 (53.7)	0.046
Lentigo	6 (5.6)	2 (3.0)	
SUM	0 (0.0)	6 (9.0)	0.006
NMN	60 (56.1)	23 (34.3)	0.005
Compound nevus	7 (11.7)	15 (65.2)	<0.001
Junctional nevus	41 (68.3)	2 (8.7)	<0.001
Intradermal nevus	12 (20.0)	6 (26.1)	

**Table 4:** Details of six SUM cases.

Patient	Sex	Age (years)	Duration	Location	Width (mm)
Xiang	Female	51	3 months	Left hallux	8
Chen	Female	49	5 months	Left thumb	Whole nail
Zhang	Male	87	20 years	Right thumb	12
Liu	Male	44	2 years	Left ring finger	10
Han	Male	55	20 years	Left thumb	12
Tang	Female	44	15 years	Left index finger	3

### Prognosis and appearance

**Figure 2:**
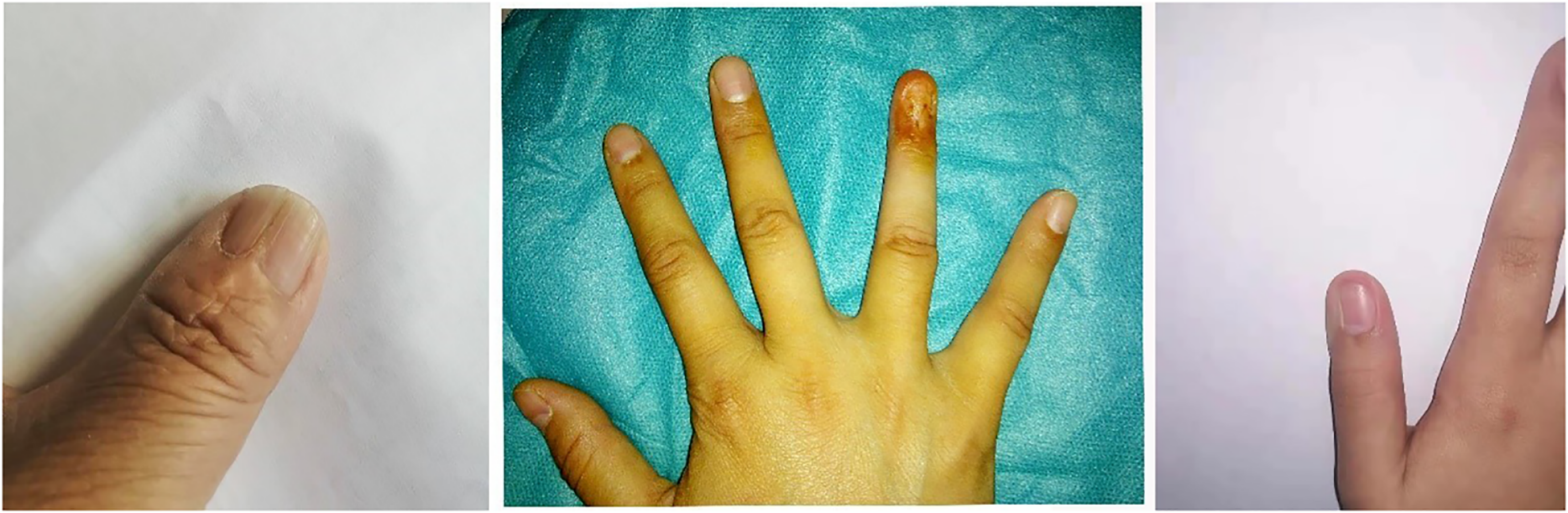
Left, nail deformity; middle, scar healing; right, nail reduction.

Nineteen patients (10.9%) recurred visible bands, 7 (6.5%) in the children’s group and 12 (17.9%) in the adults’ group. The recurrence rate in adults was significantly higher than in children (*χ*^2^ = 5.474, *p =* 0.019). The appearance variations mainly included nail reduction, nail deformity, and no nail (Figure 2). The width and location of bands are correlated closely with the outcome. When the band is in the middle, the remaining nail pieces would be sutured to shrink the wound. Removal of the nail and nail dystrophy can lead to a scar. If the lesion is close to the side, the remaining nail plate and nail bed will be sutured to the skin. A new nail groove will form, and the nail will become narrower.

## Discussion

Overall, LM is attributed to melanocytic activation, freckles, nevus, and melanoma (1). Exogenous pigments can cause black nails, and the bands are generally irregular and nonlinear (9). The diagnosis of LM is challenging due to various causes. The treatments of LM depend on the etiology and the patient. Most patients do not like to see a band on their fingers/toes. There is no consensus on the need for biopsy to rule out melanoma in children with LM (14–16). The notion that LM is benign in children is worrisome (17). The correlation analysis between the course and width is found to be positive (*r* = 0.474), which indicates that LM is a progressive disease.

The answers for benign lesions are wait-and-see or excision. Pigmentation does not require any treatment; even if removed, it often recurs. Some studies reported that two LMs recovered spontaneously in children (12, 13), which may be pigmentation based on pathological analysis. It has been reported that lentigo is prone to malignant transformation in Europe and the USA. There are only a few lentigos in this study and no canceration.

SUM is an atypical melanoma with a high incidence in Asia (5, 10). There are six cases in the adults’ group that need our attention. Assessing the regularity of lines is subjective, and early-stage SUM may appear regular. Dermoscopy may provide useful information but is not a substitute for histopathology. There is no way to distinguish them without pathology. Histopathological diagnosis of early SUM is considered very difficult, and false-positive and false-negative results have been achieved in the biopsy. A biopsy is a primary treatment for bands above 4 mm, and recurrence is the main disadvantage in 70% of cases (8). The nails are deformed after biopsy, which affects the appearance. Direct pathological examination after resection is more appropriate. After excision, reconstruction and repair, as described in this study, can improve the appearance maximally. Surgical treatment for in situ SUM removes the nail unit with phalange preservation (21). The defect can be repaired by a skin graft (22). The procedure results in good cosmetic and functional outcomes, and the prognosis is not changed (23). However, amputation is the best option for invasive SUM, preserving the functionality as more as possible (12).

NMN is the most difficult for deciding upon action. Wait-and-see is unacceptable due to the possibility of malignancy. Despite the fact that nail melanoma is rare in children, it does exist. Most NMN lesions first appearing in childhood are junctional nevi (11). In this study, 56.1% of children and only 34.3% of adults had NMN. No intervention may not benefit children with NMN (24, 25). This study shows that children have a lower recurrence rate. We believe that when children are able to cooperate with surgery, active intervention should be performed. Narrower bands preserve more nails and retain as much functionality as possible. Furthermore, some NMN lesions may be underestimated when nevus cells are neglected. The difficulty in diagnosing NMN lies in the early detection of SUM, which requires both clinical features and histopathological characteristics (18).

Therefore, physicians need to increase awareness of resection in NMNs, especially the potential malignancy. Adult NMN may become malignant after biopsy, and extended longitudinal resection is recommended. Imperfect nails are considered better than the risk of malignancy. Self-healing is possible in children, and we need more clinical studies to find the probability.

In summary, when adults find that the lesion color deepens or the width becomes wider in the short term, the lesion needs to be resected immediately. This study has some limitations (e.g., insufficient sample size and being a retrospective study).

## Significance

Different from previous methods (dermatoscopy mode), we qualitatively analyzed the pathological types to study LM from another angle. Our study highlighted important pathological differences between children and adults. The overwhelming majority of pediatric cases can be managed conservatively. Lesions in some adults may become malignant and develop into tumors. This requires different treatment options. It is beneficial for LM patients. Under this principle, patients will have a better prognosis with lower risk.

## Data Availability

The original contributions presented in the study are included in the article; further inquiries can be directed to the corresponding author.

## References

[1] Dominguez-Cherit J Roldan-Marin R Pichardo-Velazquez P Valente C Fonte-Avalos V Vega-Memije ME Melanonychia, melanocytic hyperplasia, and nail melanoma in a hispanic population. J Am Acad Dermatol. (2008) 59(5):785–91. 10.1016/j.jaad.2008.07.012 18804895

[2] Braun RP Baran R Le Gal FA Dalle S Ronger S Pandolfi R Diagnosis and management of nail pigmentations. J Am Acad Dermatol. (2007) 56(5):835–47. 10.1016/j.jaad.2006.12.021 17320240

[3] Tosti A Piraccini BM de Farias DC. Dealing with melanonychia. Semin Cutaneous Med Surg. (2009) 28(1):49–54. 10.1016/j.sder.2008.12.004 19341943

[4] Haneke E Baran R. Longitudinal melanonychia. Dermatol Surg. (2001) 27(6):580–4. 10.1097/00042728-200106000-00014 11442597

[5] Tosti A Baran R Piraccini BM Cameli N Fanti PA. Nail matrix nevi: a clinical and histopathologic study of twenty-two patients. J Am Acad Dermatol. (1996) 34(5):765–71. 10.1016/s0190-9622(96)90010-9 8632071

[6] Goettmann-Bonvallot S Andre J Belaich S. Longitudinal melanonychia in children: a clinical and histopathologic study of 40 cases. J Am Acad Dermatol. (1999) 41(1):17–22. 10.1016/S0190-9622(99)70399-3 10411404

[7] Ruben BS. Pigmented lesions of the nail unit: clinical and histopathologic features. Semin Cutaneous Med Surg. (2010) 29(3):148–58. 10.1016/j.sder.2010.06.008 21051008

[8] High WA Quirey RA Guillen DR Munoz G Taylor RS. Presentation, histopathologic findings, and clinical outcomes in 7 cases of melanoma in situ of the nail unit. Arch Dermatol. (2004) 140(9):1102–6. 10.1001/archderm.140.9.1102 15381551

[9] Cooper C Arva NC Lee C Yélamos O Obregon R Sholl LM A clinical, histopathologic, and outcome study of melanonychia striata in childhood. J Am Acad Dermatol. (2015) 72(5):773–9. 10.1016/j.jaad.2015.01.010 25766363

[10] Ohn J Choe YS Mun JH. Dermoscopic features of nail matrix nevus (NMN) in adults and children: a comparative analysis. J Am Acad Dermatol. (2016) 75(3):535–40. 10.1016/j.jaad.2016.03.043 27177439

[11] Theunis A Richert B Sass U Lateur N Sales F Andre J. Immunohistochemical study of 40 cases of longitudinal melanonychia. Am J Dermatopathol. (2011) 33(1):27–34. 10.1097/DAD.0b013e3181e67c87 20940616

[12] Banfield CC Dawber RPR. Nail melanoma: a review of the literature with recommendations to improve patient management. Br J Dermatol. (1999) 141(4):628–32. 10.1046/j.1365-2133.1999.03099.x 10583108

[13] Jung HJ Kweon SS Lee JB Lee SC Yun SJ. A clinicopathologic analysis of 177 acral melanomas in Koreans relevance of spreading pattern and physical stress. JAMA Dermatol. (2013) 149(11):1281–8. 10.1001/jamadermatol.2013.5853 24067997

[14] Takematsu H Obata M Tomita Y Kato T Takahashi M Abe R. Subungual melanoma—a clinicopathologic study of 16 Japanese cases. Cancer. (1985) 55(11):2725–31. 10.1002/1097-0142(19850601)55:11<2725::AID-CNCR2820551134>3.0.CO;2-V 3995482

[15] Feibleman CE Stoll H Maize JC. Melanomas of the palm, sole, and nailbed—a clinicopathologic study. Cancer. (1980) 46(11):2492–504. 10.1002/1097-0142(19801201)46:11<2492::AID-CNCR2820461130>3.0.CO;2-J 7438021

[16] Chi ZH Li SM Sheng XN Si L Cui CL Han M Clinical presentation, histology, and prognoses of malignant melanoma in ethnic Chinese: a study of 522 consecutive cases. Bmc Cancer. (2011) 11(10):85. 10.1186/1471-2407-11-85 21349197 PMC3056833

[17] Saida T Ohshima Y. Clinical and histopathologic characteristics of early lesions of subungual malignant-melanoma. Cancer. (1989) 63(3):556–60. 10.1002/1097-0142(19890201)63:3<556::AID-CNCR2820630326>3.0.CO;2-Q 2912531

[18] Banfield CC Redburn JC Dawber RPR. The incidence and prognosis of nail apparatus melanoma. A retrospective study of 105 patients in four English regions. Br J Dermatol. (1998) 139(2):276–9. 10.1046/j.1365-2133.1998.02365.x 9767242

[19] Tan KB Moncrieff M Thompson JF McCarthy SW Shaw HM Quinn MJ Subungual melanoma—a study of 124 cases highlighting features of early lesions, potential pitfalls in diagnosis, and guidelines for histologic reporting. Am J Surg Pathol. (2007) 31(12):1902–12. 10.1097/PAS.0b013e318073c600 18043047

[20] Amin B Nehal KS Jungbluth AA Zaidi B Brady MS Coit DC Histologic distinction between subungual lentigo and melanoma. Am J Surg Pathol. (2008) 32A(6):835–43. 10.1097/PAS.0b013e31815c8578 18391745

[21] Moehrle M Metzger S Schippert W Garbe C Rassner G Breuninger H. “Functional” surgery in subungual melanoma. Dermatol Surg. (2003) 29(4):366–74. 10.1046/j.1524-4725.2003.29087.x 12656815

[22] Lazar A Abimelec P Dumontier C. Full thickness skin graft for nail unit reconstruction. J Hand Surg-Br Eur. (2005) 30B(2):194–8. 10.1016/J.JHSB.2004.11.006 15757774

[23] Sureda N Phan A Poulalhon N Balme B Dalle S Thomas L. Conservative surgical management of subungual (matrix derived) melanoma: report of seven cases and literature review. Br J Dermatol. (2011) 165(4):852–8. 10.1111/j.1365-2133.2011.10477.x 21812768

[24] Iorizzo M Tosti A Di Chiacchio N Hirata SH Misciali C Michalany N Nail melanoma in children: differential diagnosis and management. Dermatol Surg. (2008) 34(7):974–8. 10.1111/j.1524-4725.2008.34191.x 18384604

[25] Tosti A Piraccini BM Cagalli A Haneke E. In situ melanoma of the nail unit in children: report of two cases in fair-skinned Caucasian children. Pediatr Dermatol. (2012) 29(1):79–83. 10.1111/j.1525-1470.2011.01481.x 21575049

