# Corrigendum: Retrospective analysis of longitudinal melanonychia: a Chinese experience

**DOI:** 10.3389/fped.2023.1241059

**Published:** 2023-07-11

**Authors:** Anqi Lyu, Yinglong Hou, Qiying Wang

**Affiliations:** ^1^ Department of Dermatology, Xiang'an Hospital of Xiamen University, School of Medicine, Xiamen University, Xiamen, China; ^2^ Department of Plastic Surgery, The First Affiliated Hospital of Zhengzhou University, Zhengzhou, China

**Keywords:** hutchinson’s sign, longitudinal melanonychia, nail matrix nevus, subungual melanoma, retrospective study

A Corrigendum on Retrospective analysis of longitudinal melanonychia: a Chinese experience By Lyu A, Hou Y and Wang Q. (2023) Front. Pediatr. 10:1065758. doi: 10.3389/fped.2022.1065758

**Incorrect Affiliation**

In the published article, there was an error in affiliation 1. Instead of “Department of Dermatology, Xiang’an Hospital of Xiamen University, Xiamen University, Xiamen, China”, it should be “Department of Dermatology, Xiang’an Hospital of Xiamen University, School of Medicine, Xiamen University, Xiamen, China”.

The authors apologize for this error and state that this does not change the scientific conclusions of the article in any way. The original article has been updated.

